# Enhanced atmospheric oxidation toward carbon neutrality reduces methane’s climate forcing

**DOI:** 10.1038/s41467-024-47436-9

**Published:** 2024-04-11

**Authors:** Mingxu Liu, Yu Song, Hitoshi Matsui, Fang Shang, Ling Kang, Xuhui Cai, Hongsheng Zhang, Tong Zhu

**Affiliations:** 1 State Key Joint Laboratory of Environmental Simulation and Pollution Control, College of Environmental Sciences and Engineering, Peking University, Beijing, 100871 China; 2 Graduate School of Environmental Studies, Nagoya University, Nagoya, Japan; 3 Laboratory for Atmosphere-Ocean Studies, Department of Atmospheric and Oceanic Science, School of Physics, Peking University, Beijing, 100871 China

**Keywords:** Atmospheric science, Climate change

## Abstract

The hydroxyl radical (OH), as the central atmospheric oxidant, controls the removal rates of methane, a powerful greenhouse gas. It is being suggested that OH levels would decrease with reductions of nitrogen oxides and ozone levels by climate polices, but this remains unsettled. Here, we show that driven by the carbon neutrality pledge, the global-mean OH concentration, derived from multiple chemistry-climate model simulations, is projected to be significantly increasing with a trend of 0.071‒0.16% per year during 2015–2100. The leading cause of this OH enhancement is dramatic decreases in carbon monoxide and methane concentrations, which together reduce OH sinks. The OH increase shortens methane’s lifetime by 0.19‒1.1 years across models and subsequently diminishes methane’s radiative forcing. If following a largely unmitigated scenario, the global OH exhibits a significant decrease that would exacerbate methane’s radiative forcing. Thus, we highlight that targeted emission abatement strategies for sustained oxidation capacity can benefit climate change mitigation in the Anthropocene.

## Abstract (web summary)

Atmospheric chemistry-climate model projections reveal an increase in global hydroxyl radical concentrations on the path toward carbon neutrality in the 21st century. This consequently benefits methane mitigation.

## Introduction

The atmospheric hydroxyl radical (OH) originates primarily from the collision of excited oxygen atom (O(^1^D)) produced by ozone (O_3_) photolysis with water vapor, and secondarily from radical recycling in the presence of nitrogen oxides (NO_x_)^1,2^. As the most important cleaning agent and oxidant in the troposphere, OH can immediately react with various trace gases, such as methane (CH_4_), volatile organic compounds (VOCs), and carbon monoxide (CO)^3–5^, and accordingly regulate their lifetimes and climate effects^6–8^. The spatiotemporal evolution of OH therefore plays a critical role in climate projections. Yet, it remains ambiguous how global OH will evolve with changing human activities in the Anthropocene^9–12^. A range of competing factors that determine OH sources and sinks (referred to as loss of OH via reaction with those trace gases) complicate the prediction of OH levels in the global troposphere^9,12–14^.

Future evolution of OH levels is probably linked to implementation of climate policies. The Paris Agreement calls for international cooperation to limit the global temperature rise below 1.5‒2.0 degrees relative to preindustrial levels^15,16^. Driven by the carbon neutrality pledges, the socio-economic development is likely transited to a clean energy-based society by the second half of 21st Century, with a phase-out of fossil fuels^17–19^. The targeted measures for carbon neutrality can meanwhile result in substantial reductions of near-term climate forcers (NTCFs) resulting from fossil fuel combustion, including aerosols, CO, NO_x_, and VOCs^20–22^. The projected reductions in NO_x_ and reactive carbon species is bound to weaken O_3_ formation on a global scale, because NO_2_ photolysis is a major source of O_3_ in the troposphere^23^. One could therefore expect that atmospheric OH levels will decline accordingly following a reduction in O_3_ and NO_x_^24–26^. However, the simultaneous decreases in CH_4_, CO, and VOCs emissions likely in part or fully balance the OH budget due to a reduction in OH sinks^27,28^, such that its future concentrations potentially remain at present day levels. In addition, the potential changes in physical climate including temperature and specific humidity would affect OH through impacts on its sources and sinks^29,30^.

Here, we analyze multiple model simulation results from Coupled Model Intercomparison Project Phase 6 (CMIP6)^31,32^ to reveal the trends in global OH concentration following divergent climate scenarios in the 21st century and to identify associated anthropogenic and natural drivers. Then, by reconstructing the decadal variability in global CH_4_ concentrations induced by OH evolution, we estimate CH_4_ radiative forcing linked to OH and draw implications for the policy-making of climate change mitigation. Our results shed light on the critical role of the future course in OH, resulting from changing human activities and physical climate driven by the carbon neutrality pledge, in shaping the CH_4_ trends and its climate forcing.

## Results

### Global OH evolution toward carbon neutrality

Our analysis begins with the model projections of the interannual variability of the global mean OH concentration and its link with key species related to OH sources and sinks within the troposphere, based on three ocean-atmosphere coupled Earth system models that participated in CMIP6, i.e., UKESM^33^, GFDL-ESM^34^, and MRI-ESM^35^ (see Methods). The model results are focused on the period of 2015‒2100 in a strong climate mitigation scenario towards sustainable development, Shared Socio-economic Pathway 1‒26 (SSP126)^36^. As a comparison, we also analyze the results in the SSP370 scenario, which represents a high-emissions scenario with continued increases in CO_2_ and CH_4_.

**Fig. 1:**
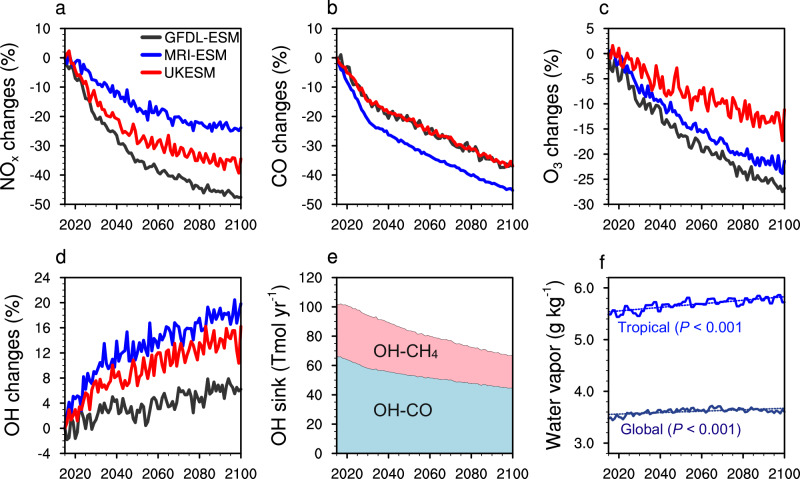
Inter-annual variability in global tropospheric hydroxyl (OH) radical and other reactive trace gases for the period of 2015‒2100. The panels illustrate the percentage changes in (**a**) nitrogen oxides (NO_x_), (**b**) carbon monoxide (CO), (**c**) ozone (O_3_), and (**d**) OH burdens compared to the corresponding 2015 levels; (**e**) Trends in OH sinks with respect to CO and CH_4_ from 2015 to 2100 derived from the ensemble mean model results following the Shared Socioeconomic Pathway 1‒2.6 (SSP126) scenario; and (**f**) Trends in global and tropical (20°S‒20°N) tropospheric mean water vapor concentrations, with the significance (*P*) shown in the right panel. The results are provided by the climate projections from three Earth system models (i.e., UKESM, MRI-ESM, and GFDL-ESM) following the SSP126 scenario, except the water vapor mixing ratios that are available only from MRI-ESM. The model diversity is illustrated by the colored lines (legend in the top left panel). We use the Mann-Kendall non-parametric test to reveal the significance of the trends for each species in the main text.

The SSP126 scenario, used here as a proxy for the carbon neutrality world, entails substantial mitigation of CH_4_ and other NTCFs from anthropogenic sources involving fossil fuel extraction, agricultural production, and landfills sectors^20,37^. The reduction in the global mean CH_4_ concentration according to the SSP126 scenario amounts to approximately 40% in total from 2015 to 2100 (Supplementary Fig. 1). Due to the rapid reduction in NO_x_ and CO emissions, the models reveal strong decreases in the tropospheric NO_x_ and CO concentrations across models (Fig. 1). These decreases in O_3_ precursors jointly weaken the formation of tropospheric O_3_, which exhibits a significantly downward trend of 0.17‒0.28% yr^−1^ (*P* < 0.001) relative to the 2015 levels (Fig. 1c). The zonal mean O_3_ decreases are widespread throughout the troposphere among three models (Supplementary Fig. 2). Interestingly, while tropospheric O_3_ concentrations decrease in the SSP126 pathway, the global OH concentrations are significantly increased, with an inter-annual trend of 0.071‒0.16% yr^−1^ (*P* < 0.001) over 2015‒2100 (Fig. 1d). Even though the simulated global OH (O_3_) burdens differ among models, they all exhibit an upward (downward) trend during the given period (Supplementary Fig. 3). By contrast, the global OH abundance in the SSP370 scenario exhibits a decreasing trend of 0.037‒0.15% yr^−1^ (*P* < 0.001) over 2015‒2100 among these models, together with an increase in tropospheric O_3_ of 0.11‒0.17% yr^−1^ (*P* < 0.001) (Supplementary Figs. 3 and 4). This decrease in OH is associated with the doubling of the CH_4_ concentration that strongly consumes OH (Supplementary Fig. 1). Overall, global OH increases with the concurrent reductions in NO_x_ and reactive carbon species during the pathway toward carbon neutrality, in contrast to the decreasing trend in an unmitigated scenario.

The projected increases in global OH under the carbon neutrality scenario (SSP126) are rooted in a range of competing factors that control the sources and sinks of OH^2,5^. Our results show an overall decline in the tropospheric annual-mean O_3_ of 11‒27% across models in the given period, with a multi-model average of 19%. Based the diagnosis of O_3_ photolysis to form excited oxygen atom in CMIP6 models (see Methods), we calculate that the OH primary production is decreased by 16‒26 Tmol yr^−1^ with the reduction of O_3_ photolysis, equivalent to 16‒22% of the 2015 levels. Note that tropospheric water vapor, through its collision with excited oxygen atom, could perturb the primary production of OH in a warmer climate^29,38^. The simulation results available in MRI-ESM show increases of tropospheric mean water vapor content by 3.8% globally and by 5.2% in the tropics (20°S‒20°N) from 2015 to 2100 in the SSP126 scenario (Fig. 1f), as a result of enhanced surface evaporation with increased temperature^2^. Given such water vapour increase, the OH production is estimated to be increased by 3.4‒4.3 Tmol yr^−1^ across models. Additionally, the OH recycling in the presence of NO_x_, as another key driver of OH (Eq. 1), is also diminished due to the pronounced decrease in NO_x_ concentration (Fig. 1a). It is estimated that the tropospheric production flux of OH through the reaction between NO and hydroperoxy radical (HO_2_) is decreased by 5.8‒20 Tmol yr^−1^. The total decrease in annual OH production dominated by O_3_ photolysis and radical recycling by NO_x_ is 28‒47 Tmol yr^−1^ from 2015 to 2100 (Supplementary Fig. 5).

1 $${{{{{{\rm{HO}}}}}}}_{2}+{{{{{\rm{NO}}}}}}\to {{{{{{\rm{NO}}}}}}}_{2}+{{{{{\rm{OH}}}}}}$$

On the other hand, we find that the total OH sink with respect to CO and CH_4_ exhibits a steadily decreasing trend (Fig. 1e), in which OH loss via the reaction with CO is greater. Under the SSP126 scenario, the reduction in CH_4_ emissions can enhance OH concentrations, shorten CH_4_ lifetimes, and amplify CH_4_ decreases, which is known as the CH_4_ self-feedback^39^. Because both CH_4_ and its oxidation products (including CO) can consume OH, the CH_4_ control contributes largely to the OH increase. Apart from these diagnosed outputs from the CMIP6 models, our estimate based on the prognostic variables including the mixing ratios of CO, CH_4_, formaldehyde, and O_3_ shows a total decrease in OH sinks of 59‒64 Tmol yr^−1^ from 2015 to 2100 (Supplementary Fig. 5). In conclusion, the reduction of OH sink consistently outweighs that of OH sources in each model projection, which accounts for the upward tendency of global OH. These results also demonstrate that the reduction of chemical loss of OH with respect to CO and CH_4_ is the leading cause of the global OH growth toward the carbon neutrality scenario, while the increase in tropospheric water vapor content may be also important.

**Fig. 2:**
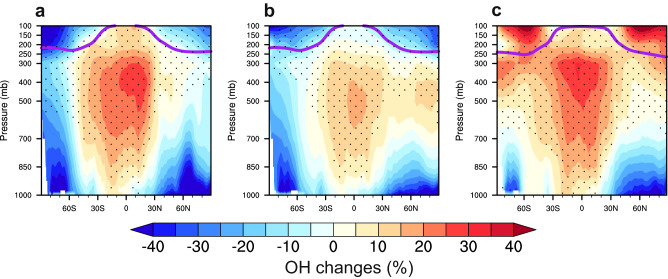
Zonal mean changes in hydroxyl radical (OH) concentrations from 2015 to 2100. The percentage changes of annual-mean OH from the three model results (**a** UKESM; **b** GFDL-ESM; **c** MRI-ESM) are presented in the zonal mean under the Shared Socioeconomic Pathway 1‒2.6 scenario. The overlaying dot pattern indicates positive shift in the ratio of NO_x_ to CO mixing ratios, used as a proxy of relative changes in OH sources and sinks. The purple solid lines denote the tropopause.

The models indicate a strong dependence of the sign and magnitude of OH changes on both latitude and altitude (Fig. 2), albeit with the overall increase in the global mean OH during the period of decarbonization. As depicted in the zonal mean distributions of OH differences in percentage, the increases in OH mixing ratios under the SSP126 scenario mainly occur in the tropical and subtropical regions, with local increases up to 30% in the free troposphere. However, the OH mixing ratio features general decreases within temperate and polar zones ( > 45 degrees), where it drops over 40% locally in the lower to middle troposphere. Because NO_x_ and CO regulate the production and loss of OH on a global scale, respectively, we employ the ratio of NO_x_ to CO mixing ratios, an indicator suggested in existing studies^8,40^, to infer the drivers of OH tendencies (marked with dots in Fig. 2). We find that increases in this ratio occur mainly in the tropics, in general coinciding with the increase in OH concentrations. As both NO_x_ and CO widely decline in the SSP126 scenario, the positive shifts in the NO_x_-to-CO ratio suggest faster decreases in CO concentrations and subsequently in OH sinks with respect to it, resulting in a net increase in OH. With regards to the large decrease of OH near the surface at the middle and high latitudes, the reduction of OH recycling via NO_x_ (R1) in response to the NO_x_ reduction and the pronounced O_3_ decreases (Supplementary Fig. 2) are the primary drivers.

### OH-mediated CH_4_ radiative forcing

**Fig. 3:**
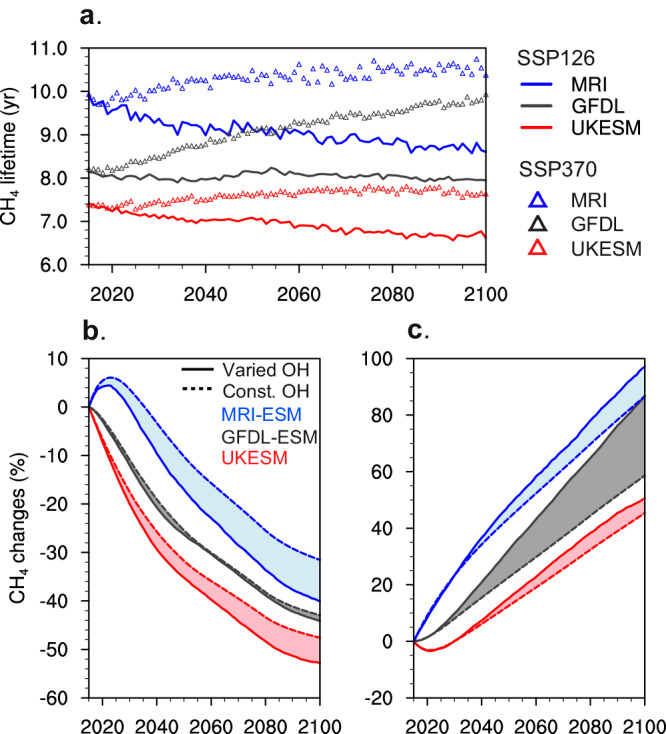
Trends in methane (CH_4_) lifetime and concentration from 2015 to 2100. **a** The diagnosed CH_4_ lifetimes in the SSP126 and SSP370 scenarios based on three models’ results. **b** The evolution of the global CH_4_ concentrations reconstructed separately with varied hydroxyl radical (OH) (solid lines) and constant OH (dashed lines) for the Shared Socioeconomic Pathway 1‒2.6 scenario; (**c**) the same with (**b**) but for the Shared Socioeconomic Pathway 3‒7.0 scenario. The effect of temperature changes is also included in our calculation. The comparison of modeled CH_4_ trends with the CMIP6 prescribed data is shown in Supplementary Fig. 7.

One of the most important implications of increasing OH on the path to carbon neutrality is its potential impact on CH_4_’s climate effect through changes in CH_4_ concentrations. We first diagnose the whole-atmosphere chemical lifetime of CH_4_ by dividing the total CH_4_ burden by the integrated loss flux over the whole model domain by referring to previous studies^9,29^. The trends in total CH_4_ lifetime are then estimated by additionally including the lifetime with respect to soil uptake of 150 years^41^. It can be seen that the lifetime trends are closely linked to those divergent OH trends (Fig. 3a). Specifically, with the increase in OH following the SSP126 scenario, the chemical loss of CH_4_ is enhanced and consequently its lifetime (a multi-model mean plus spread) is shortened from 8.5 (7.4‒9.9) year in 2015 to 7.8 (6.6‒8.8) year in 2100. Spatially, the variation of CH_4_ loss rates also tracks that of OH concentrations in the global troposphere (Supplementary Fig. 6). In line with the spatial patterns of OH changes, the CH_4_ chemical loss rates are enhanced locally by over 20% in the tropics, while reduced as much as 40% over the high latitudes.

Along with the OH changes, atmospheric temperature increases, i.e., global warming, can also shorten the CH_4_ lifetime by boosting the reaction rate of CH_4_ with OH. In our examined case, i.e., SSP126 (low forcing scenario), the global-mean surface temperature increases from 2015 to 2100 are 0.53 K‒1.6 K across models. Using the grid-resolved temperature changes from 2015 to 2100 and the CH_4_ and OH concentrations from the CMIP6 outputs (see Methods), we estimate that the CH_4_ lifetimes are shortened by 0.15‒0.27 years due to temperature increases alone. The corresponding estimates due to OH are 0.53‒1.4 years. The OH increase is the dominant driver for reductions in CH_4_ lifetimes in this scenario, while the temperature increase is also important.

In contrast, the CH_4_ lifetime is prolonged by 0.43‒1.7 years in total in the SSP370 scenario, due primarily to the decline in global OH. Compared to the SSP126 case, the larger temperature and water vapor increases under accelerated global warming partly masks the longer lifetime induced by the CH_4_ self-feedback. We estimate that in this high forcing scenario, the temperature increases shorten the CH_4_ lifetimes by 0.74‒1.1 years from 2015 to 2100, and the OH primary production is enhanced by about 25% due to water vapor increases. It has been suggested that CH_4_ increases induce a negative climate feedback on itself via global warming^29,30^.

We next identify the degree to which the CH_4_ lifetime changes dominated by OH increases can shape the CH_4_ trends in the SSP126 scenario. The global mean CH_4_ concentrations are reconstructed with the inter-annual emission data and varying lifetimes using a theoretical box model (see Methods). The lifetime effects can then be isolated by comparing the CH_4_ estimate using varying CH_4_ lifetime with that using constant lifetime (fixed at 2015). Dependent on the inter-model diversity in CH_4_ lifetimes, the changes in CH_4_ concentrations calculated using constant lifetime range from −31% to −43% between 2015 and 2100 (dashed lines in Fig. 3b). The reductions are further amplified by introducing the effects of increased OH and temperature, which together shorten the CH_4_ lifetime and lower CH_4_ concentrations in this scenario. We estimate that CH_4_ mixing ratios are further reduced by 22 ppb to 159 ppb with decreasing lifetimes (solid lines in Fig. 3b), equivalent to 2.1% to 13% relative to the 2015 levels. In contrast, the prolonged lifetime of CH_4_ in the SSP370 scenario translates into an increase of CH_4_ concentrations by 3.6% to 18% relative to the 2015 levels (Fig. 3c), in addition to those changes induced directly from the increased CH_4_ emissions. These results demonstrate that the OH evolution and its impacts on CH_4_ lifetime can markedly alter the global CH_4_ concentration by the year 2100.

Note that the CMIP6 prescribed CH_4_ concentrations were derived from the reduced-complexity climate simulations, which roughly consider the OH evolution and resulting CH_4_ lifetime changes^42^. That predicted CH_4_ decrease by 2100 for the SSP126 scenario is generally lower than our estimates of CH_4_ concentrations using the same CH_4_ emissions but with diagnostic lifetimes from the complex climate models (Supplementary Fig. 7). As detailed atmospheric chemical processes are lacked in the reduced-complexity climate model, the derived CH_4_ trends used for CMIP6 projections may need to be revisited. Moreover, the concentration-driven mode to simulate CH_4_ in CMIP6 models cannot reflect the dynamic responses of CH_4_ to tropospheric chemistry in their own experiments. Since those models simulate a wide range of OH values and corresponding CH_4_ lifetimes (Fig. 3a), the inferred CH_4_ emissions will differ in the future. We show that the declining trends in CH_4_ burdens are different among models; those with higher OH levels (shorter CH_4_ lifetimes) have larger percent decreases in CH_4_ concentrations during 2015–2100 (Fig. 3b). The more realistic changes in CH_4_ can be obtained from the emission-driven simulation for CH_4_, which enables a full coupling of CH_4_-CO-OH in the atmosphere and allows CH_4_ concentrations freely evolves^43^. Providing the decreasing CH_4_ emissions, the OH enhancement and resulting CH_4_ lifetime changes in the emission-driven simulations will be greater than presented in the prescribed data. The next phases of CMIP are encouraged to carry out the inter-model comparison of CH_4_ simulations using the emission-driven mode.

**Fig. 4:**
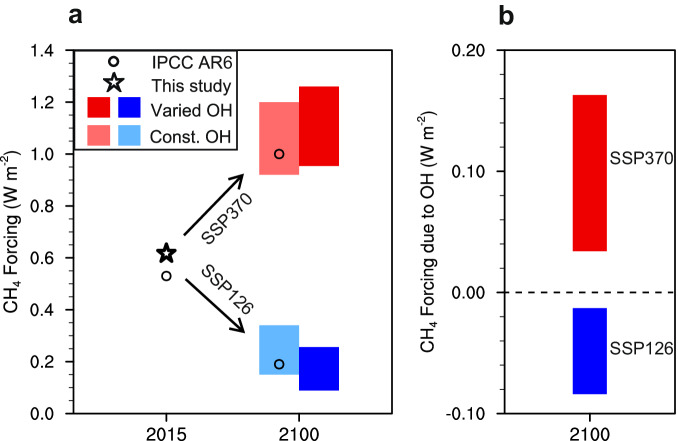
CH_4_ radiative forcing mediated by hydroxyl radical (OH) variations from 2015 to 2100. **a** CH_4_ forcing at 2015 and 2100 following the Shared Socioeconomic Pathway 1‒2.6 (SSP126) scenario (blue and light blue bars represent the intermodal diversity of CH_4_ forcing estimated using varied OH and constant OH, respectively) and the Shared Socioeconomic Pathway 3‒7.0 (SSP370) scenario (red and light red bars). **b** The contributions of OH variations on CH_4_ forcing from 2015 to 2100. The effect of temperature changes is combined with the OH effect on CH_4_ in our calculation. The positive value means the decrease in CH_4_ forcing by the OH change. The asterisk marks our estimate of CH_4_ forcing at 2015 and the black circles mark the values of CH_4_ effective radiative forcing reported by the Sixth Assessment Report of the Intergovernmental Panel on Climate Change (IPCC AR6).

Radiative forcing (RF) is a critical index that measures the change in the Earth radiative budget due to an imposed perturbation, such as the emission of anthropogenic greenhouse gases and other NTCFs, and can represent the contributions of different drivers on climate warming^44^. We calculate the temporal variations of global-mean CH_4_ RF in the 21st century using the reconstructed global CH_4_ (see Methods) and then evaluate the associated OH effects by comparing the case with varied lifetime to that with constant lifetime (fixed at 2015). Our calculation of the present-day (2015) CH_4_ RF is 0.62 W m^−2^, lying in the confidence range given in the IPCC AR6 report^45^. Adding the OH effects (along with temperature changes) on CH_4_ evolution results in more negative changes in CH_4_ RF by 2100 for the SSP126 scenario and more positive changes for the SSP370 (Fig. 4a). Specifically, due to the reductions in CH_4_ emissions in the SSP126 scenario, the CH_4_ RF in 2100 is estimated to be 0.15 to 0.34 W m^−2^ with constant OH, but decreases further to 0.089 to 0.26 W m^−2^ with increased OH and shortened CH_4_ lifetime. Thus, the net effects on CH_4_ RF are −0.013 to −0.084 W m^−2^ in 2100 among models, equivalent to 6.2% to 41% of the absolute CH_4_ RF when excluding the lifetime change, while they turn to be +0.034 to +0.16 W m^−2^ for the SSP370 (Fig. 4b). These results suggest that the increase in the OH level on the path to carbon neutrality results in an appreciable cooling effect on climate via enhanced CH_4_ removal; however, under a heavy emission scenario, the CH_4_ forcing is amplified by decreased OH.

## Discussion

Our findings suggest that the expectation that considerable NO_x_ reductions by climate polices would make OH lower in the coming decades may not be true. We identify a range of competing factors that alter OH and demonstrate that the strong decreases in global CO and CH_4_ during a pathway to carbon neutrality can diminish OH sinks and consequently result in a net increase in OH concentration from 2015 to 2100, despite a significant decrease in O_3_. This nonlinear coupling of CO-O_3_-OH-CH_4_ driven by changing human activities regulates OH atmospheric abundance throughout the 21st century. The changes in atmospheric temperature and water vapour concentrations under a warmer climate also participate in this chemical coupling. Such future trajectory towards carbon neutrality has the added benefit for CH_4_ mitigation.

Though the world pledges to reach net-zero carbon emissions in the middle of the century, it is still a challenge to deliver on this pledge; due to this, anthropogenic emissions might not perfectly follow the predefined climate scenarios. Their long-term trends will depend on the degree to which targeted climate polices as well as air pollution control measures can be implemented both locally and globally. Specifically, CH_4_ emission regulation remains a tricky problem. The recent surge in atmospheric CH_4_ growth rates emphasizes the importance of reinforcing control on CH_4_ from anthropogenic sources. CH_4_ is emitted not only from oil and gas industry, but also from agricultural production and landfills. In the unmitigated scenario, SSP370, the agricultural sector will become the dominant emitter of CH_4_ in the second half of the 21st century. We find that the rapidly increased CH_4_ concentration in this case would induce a decrease in OH, which then accelerates the rise of CH_4_ due to the positive CH_4_ self-feedback on its lifetime and amplifies CH_4_ radiative forcing by up to 0.16 W m^−2^.

Our study highlights the importance of accounting for the changes in the atmospheric oxidizing capacity when formulating climate policies to control greenhouse gas emissions, as the concomitant reductions of short-lived trace species, including NO_x_ and reactive carbon species, can collectively determine OH concentrations and thus impact CH_4_ budgets. A sustained OH level, achieved by a delicate balance between OH sources and sinks, is beneficial for climate change mitigation in the pathway toward a carbon neutral world.

## Methods

### CMIP6 projections

Three state-of-the-art Earth system models that participated in the AerChemMIP CMIP6 experiments^46^ are analyzed for OH tendencies: Geophysical Fluid Dynamics Laboratory Earth System Model version 4 (GFDL-ESM)^34^, United Kingdom Earth System Model version 1.0 with low resolution (UKESM)^33^, and the Meteorological Research Institute Earth System Model Version 2 (MRI-ESM)^35^ (Supplementary Table 1). These Earth system models with coupled atmosphere-ocean configuration are capable of representing the roles of both anthropogenic and natural drivers in OH trends under a warmer climate. All these models are configured with detailed interactive atmospheric chemistry and aerosols for the troposphere and stratosphere, involving the chemical system for O_x_–HO_x_–NO_x_–CH_4_–CO reactions and NMVOC oxidation.

In this study, we focus on the period of 2015‒2100 using the transient CMIP6 simulations in future scenarios. Designed for the achievement of the carbon neutrality in the world by the second half of the 21st century to limit the global temperature rise within 2.0 degree, preferably below 1.5 degree, one commonly-adopted climate scenario, SSP1-26 scenario, is chosen for analysis among various possible socioeconomic futures^16,36^. The SSP1-26 represents the green road with a global shift toward a more sustainable future, aimed to achieve the radiative forcing target at 2.6 W m^−2^ over the course of century to meet the UN Paris Agreement. Following this scenario, Global CO_2_ emission will reach zero threshold around 2070, and emissions of aerosols and trace gases like sulfur dioxide and nitrogen oxides from energy and industrial sectors decline sharply due to extensive use of non-fossil fuel energy and end-of-pipe measures.

As a comparison, we examine the OH evolution and its effects on CH_4_ in an unmitigated climate scenario with no effective climate policies carried out, called SSP3-70, which represents a high radiative forcing of 7.0 W m^−2^ with carbon emission continuing to rise throughout the 21st century. Compare to other SSP scenarios, SSP3-70 shows the highest emissions of methane and other near-term climate forcers at 2100, equivalent to or exceeding the present-day levels. Of them, anthropogenic methane emission exhibits a steadily increasing trend with the 2100 level almost doubling that of 2015^20^. Overall, the SSP1-26 and SSP3-70, as two established scenarios with divergent emission trajectories, can provide a robust experimental platform to study atmospheric chemistry-climate interactions and to inform optimum climate policy formulation.

From CMIP6 models, the CH_4_ simulations are driven by prescribed surface concentrations as lower-boundary conditions from present day to the future constructed following different scenarios. These future CH_4_ concentrations have been projected in support of CMIP6 using a much simplified climate-carbon-cycle model without the consideration of evolution of natural CH_4_ emissions in response to climate warming^42^. A more physically-based method is to model CH_4_ concentrations online with emission fluxes, i.e., the emission-driven mode, which allows a fully interactive, process-level simulation of the CH_4_ budget and its response to climate change^47^, but this is beyond the scope of this study. The current CMIP6 simulations are able to reflect the changes in global OH in relation to evolving CH_4_ concentrations given in different scenarios, but their results are not fully self-consistent, as each model simulation infers its own CH_4_ trajectory that typically differs from the prescribed CH_4_ trajectory for the scenario.

Of CMIP6 ensemble simulations, we chose ensemble member “r1i1p1f1” for GFDL-ESM and MRI-ESM and “r1i1p1f2” for UKESM. All models provide monthly results of atmospheric mixing ratios of various trace species including OH, CH_4_, O_3_ and NO_2_, which are averaged in our analyses to obtain annual means. The diagnosed CH_4_ loss rates in each grid box that are controlled predominantly by the OH-CH_4_ reaction are extracted. Then, we can derive the global-mean CH_4_ lifetime by combining its whole-atmosphere integrated loss fluxes and the global total CH_4_ burden. We also calculate the normalized CH_4_ loss rate (yr^−1^) by dividing the CH_4_ loss by the CH_4_ burden.

In the CMIP6 archive, the loss fluxes of CH_4_ and CO with OH are provided, which are used to show the changes in OH sinks, while the OH production fluxes are not available from the CMIP6 outputs. To enable the comparison of the changes between the OH sources and sinks, we combine the modeled trace gas mixing ratios and diagnosed O_3_ photolysis rates to estimate the production and loss fluxes of OH in the troposphere. Based on the availability of CMIP6 data, we consider three source terms (O(^1^D) + H_2_O, NO + HO_2_, and O_3_ + HO_2_) and six sink terms (the reaction of OH with CH_4_, CO, O_3_, HO_2_, HCHO, and NO_2_). It has been suggested that based on steady-state approximations, applying these terms can derive reasonable spatial and temporal information on OH at a global scale^48^. The change in water vapour concentrations is include in the calculation. The temperature dependent reaction rate coefficients are derived using the time- and grid-resolved temperature from the models. Our calculation includes a representation of the effect of global warming on the OH budget.

### Theoretical box model

The chosen CMIP6 models above can represent the effects of CH_4_ on atmospheric chemistry including OH evolution, but they are not able to simulate CH_4_ mixing ratios in the atmosphere interactively because CH_4_ concentrations in the models are constrained by the surface boundary input data. In this study, to reveal the importance of OH in driving CH_4_ evolution, we reconstruct the inter-annual variations in global mean CH_4_ concentrations in different scenarios using a simple theoretical box^49^ model expressed as the equation below:

2 $$\frac{d[{{CH}}_{4}]}{{dt}}=\frac{-[{{CH}}_{4}]}{\tau }+E$$

Where [CH_4_] denotes the mixing ratio (ppbv) of CH_4_ in the atmosphere; t denotes time in years; τ denotes CH_4_ lifetime in years calculated using the CMIP6 results and a constant lifetime (150 years) with respect to soil uptake; and E denotes the growth of CH_4_ concentration in ppb per year associated with the emission flux. The CMIP6 database provides the model outputs of CH_4_ loss fluxes, which are integrated in time and space in this study to obtain the annual total loss for each model. In line with Prather et al.^50^, we use the CH_4_ turnover lifetime, defined as the CH_4_ burden divided by the loss rate, to project future global-mean CH_4_ abundance. The lifetimes derived from transient chemistry-climate simulations consider various chemistry-climate factors in determining OH, including the CH_4_ feedback.

In the Eq. 2, the global anthropogenic and biomass burning emissions of CH_4_ are provided by the input data sets for Model Intercomparison Projects (input4MIPs) in accordance with CMIP6 SSP scenarios. Natural emission including wetland is taken from the World Data Center for Climate (WDCC) at DKRZ^51^. In this database, the natural emission at the year 2015 is 222 Tg. These long-term emissions are used to model [CH_4_] in Eq. 2 based on a constant factor of 2.75 Tg per ppbv, which is applied for the conversion from emission fluxes (Tg yr^−1^) to atmospheric mixing ratios (ppbv)^50^. We solve this differential equation to obtain [CH_4_] using an explicit method. Because CH_4_ lifetimes evolve with OH variations from 2015 to 2100, the [CH_4_] variability is dependent on both emissions and lifetime changes. We also obtain the results by fixing the lifetime at the 2015 level in Eq. 2 and compare them with those using varying OH to identify the role of OH in shaping global CH_4_ concentrations in the 21st century for SSP1-26 and SSP3-70 scenarios, respectively.

### Climate forcing estimation

Using these reconstructed CH_4_ concentrations in different cases, we then estimate the CH_4_ radiative forcing (W m^−2^) relative to preindustrial levels using the following equation^52^:

3 $${RF}\left({{CH}}_{4}\right)=\,	\left(-1.3\times {10}^{-6}\times \frac{(M+{M}_{0})}{2}-8.2\times {10}^{-6}\times \frac{(N+{N}_{0})}{2}+0.043\right) \\ 	 \times (\sqrt{M}-\sqrt{{M}_{0}})$$

Where M denote the global mean mixing ratios of CH_4_ derived from Eq. 2; N denotes the N_2_O mixing ratio simulated by the CMIP6 models; M_0_ and N_0_ denote the initial CH_4_ and N_2_O at the preindustrial (for the year 1750) level and equal to 722 ppb and 270 ppb, respectively^52^. Note that this RF is analogous to instantaneous forcing, but the former including adjustment to stratospheric temperatures. We calculate the CH_4_ climate forcing separately with and without the perturbation due to OH by 2100. The OH projections are provided for a carbon neutrality scenario (SSP126) and a heavy emission scenario (SSP370), respectively.

### Trend analysis

We perform the time series analysis for interannual mean global OH as well as other trace species by using the Mann-Kendall non-parametric test for monotonic trend. The linear trend is calculated using the Theil-Sen robust estimate.

### Supplementary information

Supplementary Information

Peer Review File

## Data Availability

The model data from the Coupled Model Intercomparison Project Phase 6 are publicly available on the Earth System Grid Federation (ESGF) website (https://esgf-data.dkrz.de/search/cmip6-dkrz/ or https://aims2.llnl.gov/search/cmip6/). The source data for reproducing the figures are available in a public repository at: 10.5281/zenodo.10784591.

## Code availability

The codes created for analyzing the CMIP6 experiments are publicly available (10.5281/zenodo.10820551).
