# Supplementary material for: Enhanced atmospheric oxidation toward carbon neutrality reduces methane’s climate forcing
Source: Nat Commun. 2024 Apr 11;15:3148. doi: 10.1038/s41467-024-47436-9 (PMC11009326; doi:10.1038/s41467-024-47436-9)
Supplement: Supplementary file 1 — Supplementary Information [file 41467_2024_47436_MOESM1_ESM.pdf]

Supplementary Information for

Enhanced atmospheric oxidation toward carbon neutrality reduces methane's climate forcing

Authors: Mingxu Liu[1,2], Yu Song[1], Hitoshi Matsui[2]*, Fang Shang[1], Ling Kang[1], Xuhui Cai[1], Hongsheng Zhang[3], Tong Zhu[1]*

[1]State Key Joint Laboratory of Environmental Simulation and Pollution Control, College of Environmental Sciences and Engineering, Peking University; Beijing 100871, China.

[2]Graduate School of Environmental Studies, Nagoya University; Nagoya, Japan.

[3]Laboratory for Atmosphere-Ocean Studies, Department of Atmospheric and Oceanic Science, School of Physics, Peking University; Beijing 100871, China.

*Corresponding author. Email: matsui@nagoya-u.jp (H.M.) and tzhu@pku.edu.cn (T.Z.).

This PDF file includes:

Supplementary Figs. 1-7

Supplementary Table 1

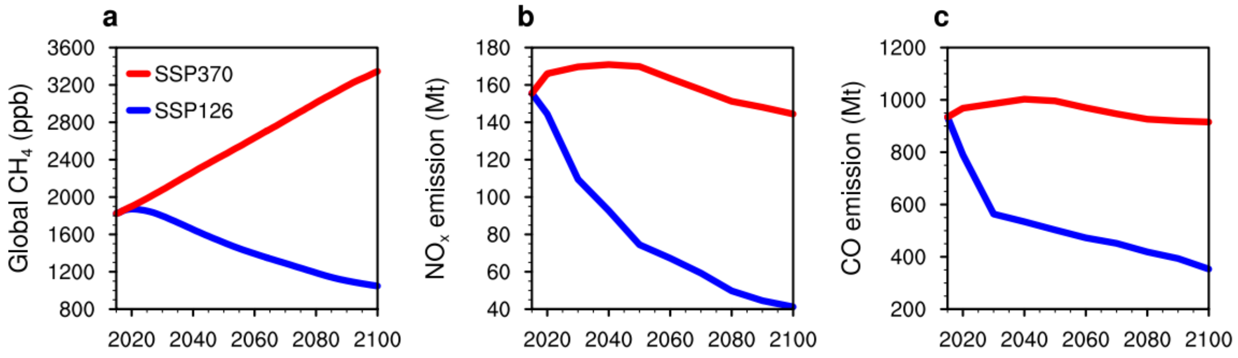

Supplementary Fig. 1 | Concentration and emissions following the SSP126 and SSP370 scenarios used in CMIP6 simulations. The panels show (a) CH_4 global annual mean concentrations from 2015 to 2100, (b) annual emission fluxes of nitrogen oxides (NO_x), and (c) emissions of carbon monoxide (CO).

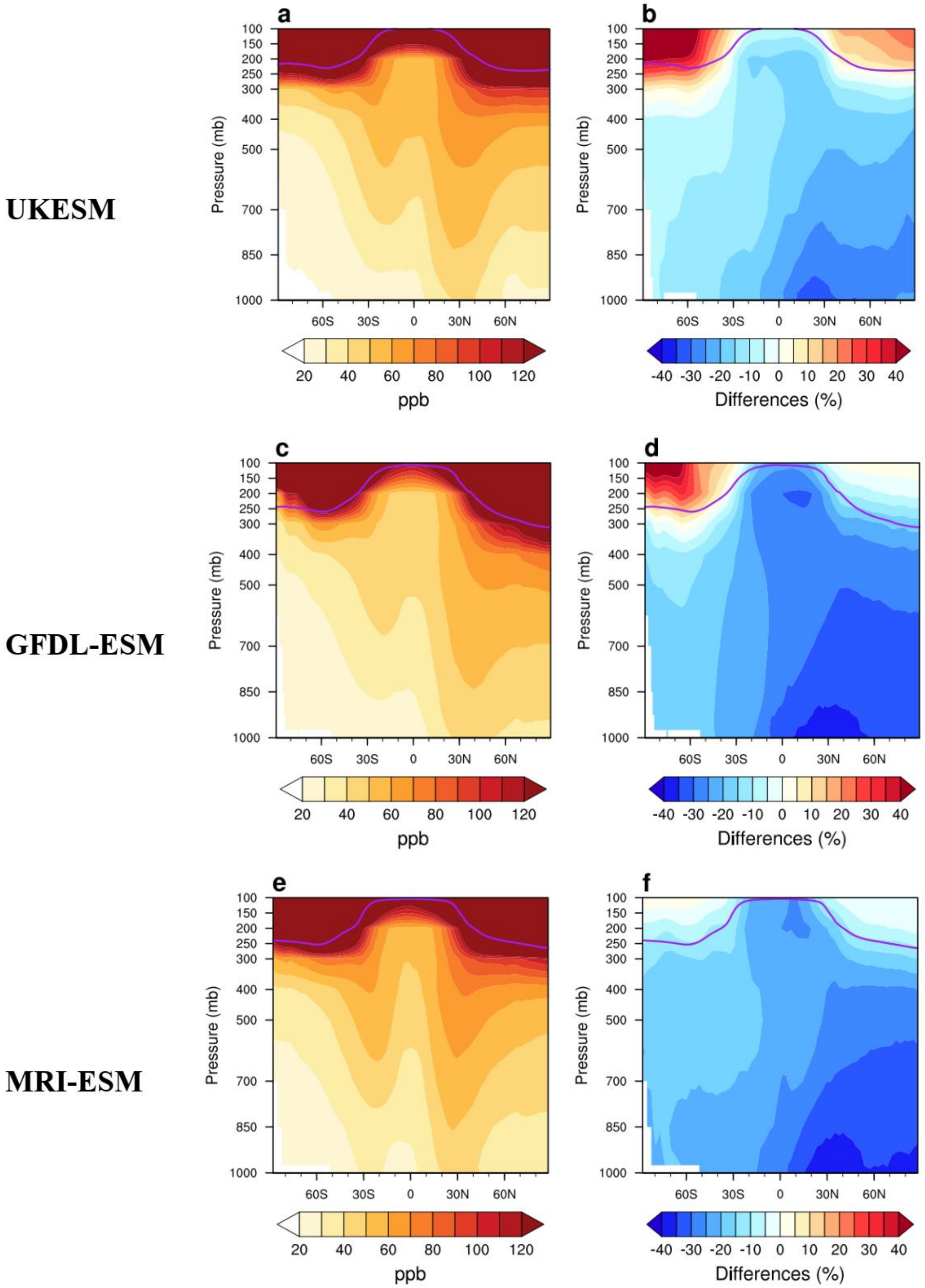

Supplementary Fig. 2 | O_3 mixing ratios and their changes from 2015 to 2100 under a carbon neutrality scenario. Zonal mean mixing ratios and percentage changes in O_3 among the UKESM (a-b), GFDL-ESM (c-d), and MRI-ESM (e-f) models (from top to bottom) in the SSP126 scenario during the 2015–2100 period. The purple solid lines denote the tropopause.

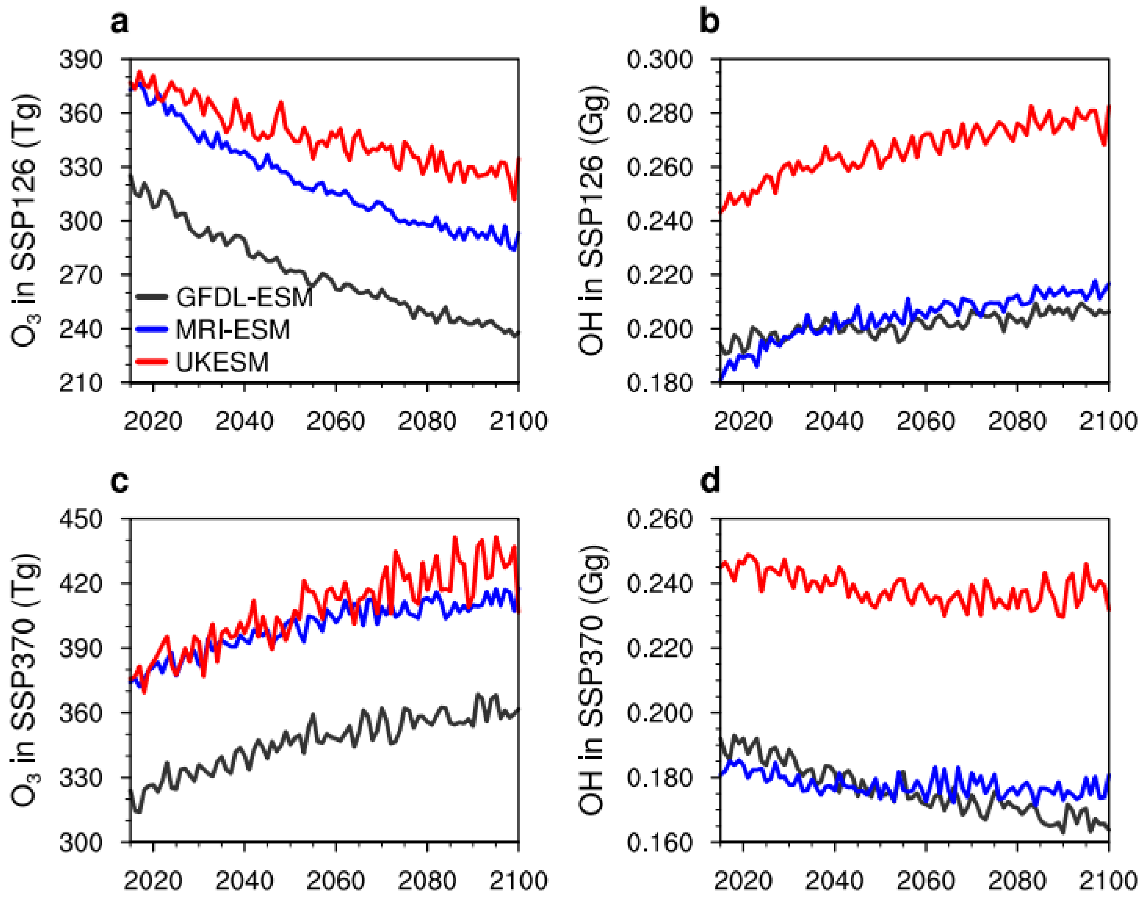

Supplementary Fig. 3 | Interannual variability in simulated tropospheric O_3 and OH concentrations from 2015 to 2100 under the SSP126 and SSP370 scenarios, respectively. Global burdens of (a, c) O_3 and (b, d) OH are derived from climate projections by three Earth system models (i.e., UKESM, MRI-ESM, and GFDL-ESM).

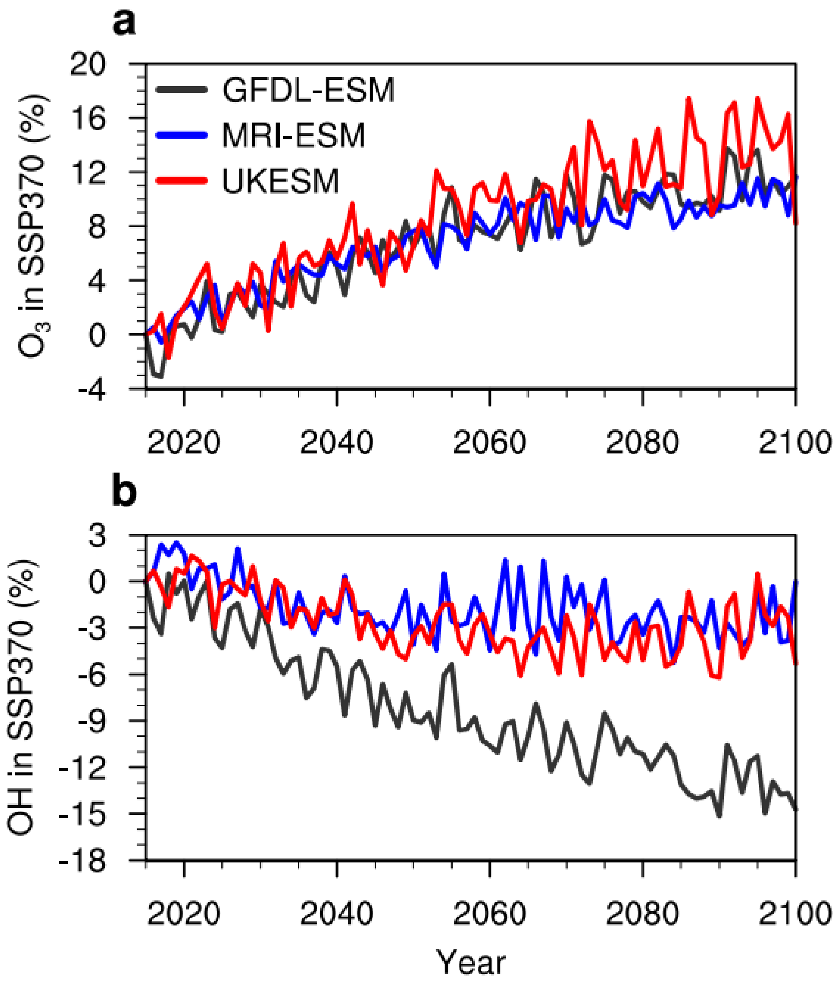

Supplementary Fig. 4 | Relative changes of simulated tropospheric O_3 and OH concentrations compared to the 2015 levels under the SSP370 scenario. Percentage changes in global (a) O_3 and (b) OH are derived from climate projections by three Earth system models (i.e., UKESM, MRI-ESM, and GFDL-ESM).

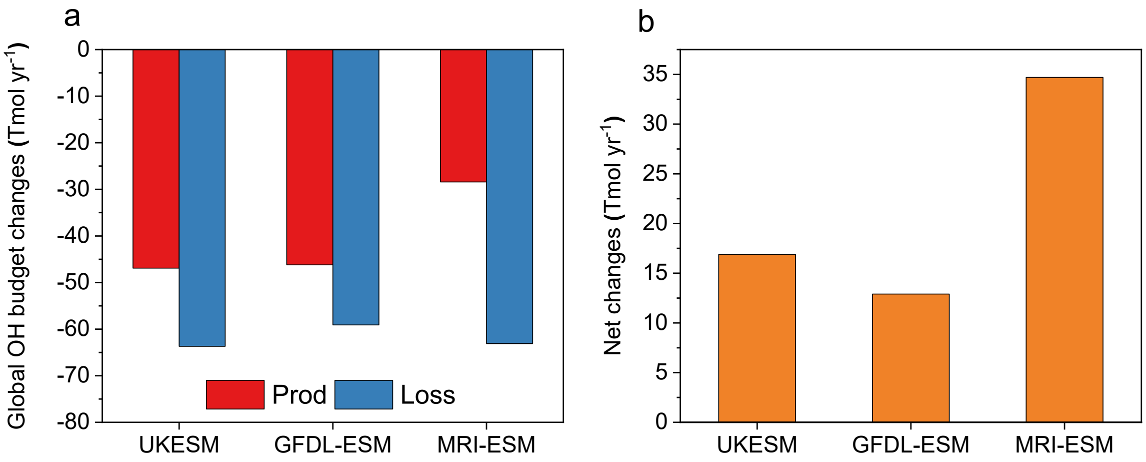

Supplementary Fig. 5 | Changes in OH production and loss fluxes from 2015 to 2100 in the SSP126 scenario. The panels show (a) the respective changes in global OH sources and sinks calculated using there CMIP6 model results and (b) the net changes in OH budgets.

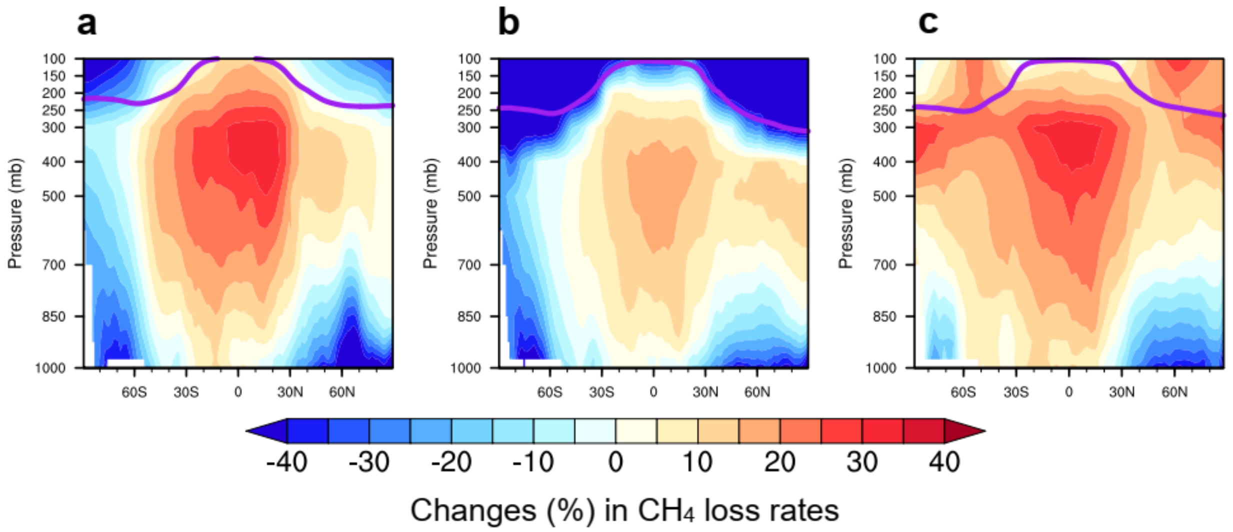

Supplementary Fig. 6 | Zonal mean changes (%) in CH_4 loss rates (yr^{-1}) derived from the UKESM, GFDL-ESM and MRI-ESM (a-c). These percentage changes were calculated for the Shared Socioeconomic Pathway 1–2.6 scenario during the period of 2015–2100. The purple solid lines denote the tropopause.

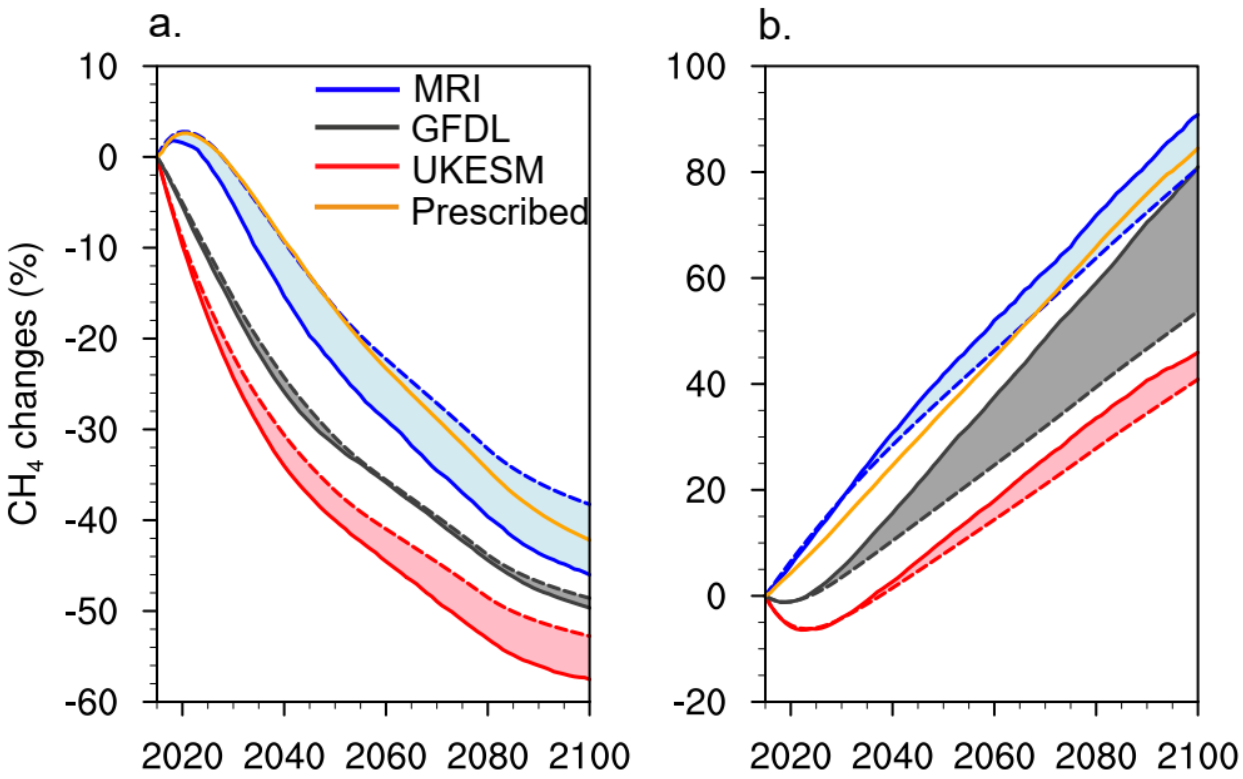

Supplementary Fig. 7 | The comparison of the CH_4 trends derived in this study with the CMIP6 prescribed data. The panels show the percentage changes relative to the 2015 levels for the (a) SSP126 scenario and (b) the SSP370 scenario. For our results, the solid and dashed lines stand for the calculation using varying OH and fixed OH, respectively. The same CH_4 emissions are used for deriving our results and the prescribed data.

Supplementary Table

Supplementary Table. 1 | CMIP6 models used in this study.

Model	Institute	Ensemble member
UKESM1-0-LL	MOHC	r1i1p1f2
GFDL-ESM4	NOAA-GFDL	r1i1p1f1
MRI-ESM2-0	MRI	r1i1p1f1
